# Anticancer effects of bifidobacteria on colon cancer cell lines

**DOI:** 10.1186/s12935-021-01971-3

**Published:** 2021-05-12

**Authors:** Zeinab Faghfoori, Mohammad Hasan Faghfoori, Amir Saber, Azimeh Izadi, Ahmad Yari Khosroushahi

**Affiliations:** 1 Food (Salt) Safety Research Center, School of Nutrition and Food Sciences, Semnan University of Medical Sciences, Semnan, Iran; 2 Department of Medical Biotechnology and Nanotechnology, School of Medicine, Zanjan University of Medical Sciences, Zanjan, Iran; 3 Department of Nutritional Sciences, School of Nutritional Sciences and Food Technology, Kermanshah University of Medical Sciences, Kermanshah, Iran; 4 Student Research Committee, Tabriz University of Medical Sciences, Tabriz, Iran; 5 Drug Applied Research Center, Tabriz University of Medical Sciences, Tabriz, Iran; 6 Department of Medical Nanotechnology, Faculty of Advanced Medical Science, Tabriz University of Medical Sciences, Tabriz, Iran; 7 Department of Nutritional Sciences, School of Nutritional Sciences and Food Technologies, Kermanshah University of Medical Sciences, Isar Sq., next to Farabi Hospital, P.O. Box 6719851351, Kermanshah, Iran

**Keywords:** Colorectal cancer, Probiotic, Bifidobacteria, Apoptosis

## Abstract

**Background:**

Colorectal cancer (CRC), with a growing incidence trend worldwide, is resistant to apoptosis and has uncontrolled proliferation. It is recently reported that probiotic microorganisms exert anticancer effects. The genus *Bifidobacterium*, one of the dominant bacterial populations in the gastrointestinal tract, has received increasing attention because of widespread interest in using it as health-promoting microorganisms. Therefore, the present study aimed to assess the apoptotic effects of some bifidobacteria species on colon cancer cell lines.

**Methods:**

The cytotoxicity evaluations performed using MTT assay and FACS-flow cytometry tests. Also, the effects of five species of bifidobacteria secretion metabolites on the expression level of anti- or pro-apoptotic genes including BAD, Bcl-2, Caspase-3, Caspase-8, Caspase-9, and Fas-R studied by real-time polymerase chain reaction (RT-PCR) method.

**Results:**

The cell-free supernatant of all studied bifidobacteria significantly decreased the survival rates of colon cancer cells compared with control groups. Flow cytometric and RT-PCR results indicated that apoptosis is induced by bifidobacteria secretion metabolites and the mechanism for the action of bifidobacteria species in CRC prevention could be down-regulation and up-regulation of anti-apoptotic and, pro-apoptotic genes.

**Conclusions:**

In the present study, different bifidobacteria species showed anticancer activity on colorectal cancer cells through down-regulation and up-regulation of anti-apoptotic and pro-apoptotic genes. However, further studies are required to clarify the exact mechanism of apoptosis induction by bifidobacteria species.

## Introduction

Cancer as a major public health problem has a growing incidence trend worldwide. It is estimated that the number of global cancer deaths rises 45% from 2007 to 2030 [1, 2]. CRC, the most common malignancy of the gastrointestinal tract, is the third and second most common cancer in males and females, respectively, and is the fourth leading cause of cancer death worldwide [3, 4]. The number of new cases is increasing quickly due to the rising of different risk factors. The risk factors for CRC are including modifiable such as smoking, alcohol, physical inactivity, and obesity, and nonmodifiable factors like familial risk, male sex, older age, and race/ethnicity [5]. It has been indicated that the general population of intestinal bacteria and microbial dysbiosis may contribute to the initiation and the etiology of CRC [6] thus, any factor that could modify the gut microbiota may prevent the disease. Some studies proposed that the ingestion of certain microorganisms decreases both the risk of developing certain types of cancer and also tumor growth, so a lot of attention has been focused on probiotic yeasts and bacteria like *lactobacilli* and bifidobacteria [7–11].

The genus *Bifidobacterium* contains approximately 57 (sub) species. They are among the dominant bacterial populations in the gastrointestinal tract and the normal inhabitants of a healthy human gut. To date, almost ten species/subspecies of *Bifidobacterium* have been detected in the human intestine. Among the bifidobacterial species, *B. catenulatum*, *B. pseudocatenulatum*, *B. adolescentis*, *B. longum*, *B. breve*, *B. bifidum*, *B. animalis*, and *B. dentium* are commonly found in the feces of healthy subjects [12, 13]. Numerous studies indicated that changes in the number or/and composition of their populations are one of the most frequent situations that present in some diseases like inflammatory bowel disease, colorectal cancer, or irritable bowel syndrome [14]. During infancy, almost 80% of the gut microbiota consisted of bifidobacteria, and dysbiosis during infancy increases the risk of childhood diseases and could also affect the health of the host in the future [15].

In the last 15 years, the genus *Bifidobacterium* has received increasing attention due to widespread interest in using as health-promoting microorganisms, i.e., known as probiotics, in the food industry, and many products containing bifidobacteria such as fermented milk, yogurt, and healthy foods that produced for the microbiota modification [16, 17].

Some species of bifidobacteria are able to decrease carcinogen-induced DNA damage, pre-neoplastic lesions, and tumors in the colon of rats [18, 19]. Administration of 3 bacteria including *Lactobacillus acidophilus*, *B. bifidum*, and *B. infantum* altered the gut microbiota and decreased colon cancer development by decreasing tumor incidence, multiplicity/count, and volume [7]. Moreover, a recent study investigated the effects of *Bifidobacterium. Breve*, *Lactobacillus. reuteri* and a cocktail of 10 strains from *Lactobacillus* and *Bifidobacterium* on LS174T (human colon adenocarcinoma cell line) and CRC mice model. In this study, treatment with Bifidobacteria showed the most apoptosis-inducing effects on LS174T cells in comparison to other bacterial treatments. Besides, the incidence of tumors and their size was significantly lower and smaller in Bifidobacteria treated mice in comparison to untreated CRC mice [20]. In another study, the anticancer effect of *B. adolescentis* SPM0212 extract, isolated from fecal samples of healthy peoples, was studied on human colon cancer cell lines (Caco-2, HT-29, and SW480) and the results showed that its extract significantly inhibits the proliferation of both cell lines [21].

The exact mechanisms responsible for the anti-cancer activity of these organisms are unknown yet, but some proposed mechanisms may influence metabolic, immunological, and protective functions within the colon, and also they may stimulate tumor cell apoptosis [22]. Apoptosis is an active cellular process that damaged or mutant cells undergo self-destruction and can help the organism for control normal development. Each step of apoptosis requires many proteins such as caspases that block tumor progression [23]. However, the exact molecular pathways by which bifidobacteria affect tumor cells remains unclear. Therefore, the present study aimed to assess the anticancer effects of some bifidobacteria species on HT-29 and Caco-2 cell lines in comparison to normal epithelial cells (KDR/293) by focusing on the main apoptosis pathways.

## Materials and methods

### Cell-free supernatant preparation

Five species of bifidobacteria including *B. adolescentis* (ATCC 15703, PTCC1536), *B. animalis subsp. lactis* (PTCC1736), *B. animalis subsp. animalis* (ATCC 25527, PTCC 1631), *B. bifidum* (ATCC 29521, PTCC 1644), and *B. ‌angulatum* (ATCC 27535, PTCC 1366) were obtained from the Persian Type Culture Collection (PTCC) from the Iranian Research Organization for Science and Technology (IROST). Bifidobacteria strains were cultured in de Man–Rogosa agar (MRS) (Merck, Darmstadt, Germany) at 37 °C for 72 h in an anaerobic incubator. Then, the cultures were centrifuged at 4500 rpm for 15 min at 4 °C. Fifty mL of each supernatant mixed with 75 mL volume of methanol and gently agitated for 24 h. The methanolic extracts were dried by lyophilization and obtained dried materials were dissolved in different amounts (5–30 mg/mL) in each used cell culture including Dulbecco Modified Eagle medium (DMEM) (Gibco, Grand Island, NY, USA), and Roswell Park Memorial Institute medium (RPMI) 1640 (Sigma, St Louis, MO, USA). Finally, the pH of supernatants was adjusted to 7.2 points and before using, filtered through a 0.22 mm Millipore filter (Milli-Q, Millipore, Germany).

### Cell culture

Two human colon cancer cell lines (Caco-2, ATCC, HTB-37 and HT-29, ATCC, HTB-38) and one human epithelial normal cell line with the same embryonic origin (KDR/293) were purchased from Pasteur Institute (national cell bank of Iran) at 13 Feb 2020. Moreover, all of the cell lines were certified recently and also tested for mycoplasma contamination by Pasteur Institute. The purchased cells were cultured in 25 cm^2^ plastic cell culture flasks and were incubated under standard conditions at 37 °C in a humidified atmosphere with 5% CO_2_ with medium renewal every 1–3 days. The cells were maintained in an RPMI-1640 (HT-29 and Caco-2) or high glucose concentration (4.5 g/L) DMEM (KDR/293) cell culture medium, respectively. Both media were supplemented with 10% (v/v) fetal bovine serum (FBS) (HyClone, Logan, UT, USA), 8mM l-‌glutamine, ‌and 1% of mixture penicillin (100 IU/mL) and streptomycin (100 g/mL) (Sigma, St Louis, MO, USA).

### MTT assay

Cell viability in treated and untreated cell lines was determined by the 3-(4,5-dimethylthiazole-2-yl)-2,5-diphenyltetrazolium bromide (MTT) assay (Sigma, St Louis, MO, USA) based on the capacity of viable cells to reduce a tetrazolium colorless salt to purple formazan in mitochondria. At first, the half-maximal inhibitory concentration (IC_50_) for HT-29 and Caco-2 cells was determined using prescreening MTT tests (in the range of 10 to 100 µg/mL) at 24 and 48 h. Briefly, the cells were washed twice with phosphate-buffered saline (PBS) (Sigma, St Louis, MO, USA) and trypsinized by adding 1 mL of trypsin/EDTA (Sigma, St Louis, MO, USA) solution. The cells were plated into 96well plates at 1.2 × 10^4^ cells per well and added 200 µL of the growth medium, incubated for 24-h. After cell attachment, the medium was carefully removed from each well and the cells were treated with an effective dose (IC_50_) of cell-free bacterial supernatant and growth medium. After 24 or 48 h (determined time point) incubation, 50 µL of MTT reagent and 150 µL of fresh growth medium were added to each well and plates returned to the incubator for 4 h. Then, the medium of each well was carefully removed and 200 µL of dimethyl sulfoxide (Merck, Germany) and 25 µL of Sorenson buffer (0.1 mol/L glycine, 0.1 mol/L NaCl, pH 10.5) were added to each well and kept for 15 min in the dark condition at room temperature. The absorbance was determined using an enzyme-linked immune-sorbent assay plate reader (ELx 800; Biotek, Winooski, VT, USA) at 570 nm. The growth inhibitory effects of supernatant were calculated according to the following formula: the growth inhibition ratio = [(the absorbance of the blank control group – the absorbance of experimental group)/the absorbance of blank control group] × 100% [24, 25]. To compare the effect of MRS, as bacteria culture media (negative control), with studied bacteria supernatants, both cancerous and normal cells were treated by methanolic extract of intact MRS media that prepared similar to bacteria supernatants. After lyophilization of methanolic extract of MRS media (50 mL), the obtained dried materials were 18 mg. Moreover, 5-fluorouracil (5-FU) as an approved anticancer drug (7 µL/well of 96-well plate) was used as the positive control.

### Flow cytometry

Three mL of growth medium including 1.2 × 10^5^ cells was cultured in 6-well culture plates and incubated at growth condition. After 24 h, the cells were treated with 3 mL of the sterile growth medium containing determined dried materials of supernatant or 5-FU, as the positive control group, and incubated in the growth condition based on the determined time point. The treated/untreated control cells were detached by trypsin-EDTA, and supernatants were discarded by centrifugation at 900 rpm for 10 min. Finally, for detection of apoptosis, the cells were stained with Annexin V-FITC/Propidium iodide (PI) apoptosis kit (eBioscience, San Diego, CA, USA) according to the manufacturer’s instructions, and data analysis was conducted using CELL Quest Pro software (BD Biosciences, San Jose, CA, USA). After performing the flow cytometry, the cell populations were defined using quadrant gates. The number of cells in each quadrant represented quadrant 1 (Q1): necrotic cells (Annexin V−/PI+); quadrant 2 (Q2): late apoptotic cells (Annexin V+/PI+); quadrant 3 (Q3): early apoptotic cells (Annexin V+/PI−); and quadrant 4 (Q4): live cells (Annexin V−/PI−) [26, 27]. Each experiment was repeated 2 times with triplicate samples. All of the analyses were performed using 150,000 cells at a rate of 900 cell/sec.

### Quantitative real-time PCR analysis

All untreated/treated cells were washed three times with PBS (pH 7.2) and total RNA was extracted from cells by direct lysis using 1 mL ice cold RNX-plus solution (Sina Clone, Iran), according to the manufacturer’s instruments. The obtained total RNA was solved in 50 µL DEPC-treated water (Merck, Germany), and the quantity and quality of total RNA were evaluated by UV spectrophotometry and agarose gel electrophoresis, respectively. Complementary DNA (cDNA) was synthesized using one microgram of isolated RNA by Prime Script RT Reagent kit (Takara Bio Inc, Tokyo, Japan) according to the manufacturer’s instructions. The specific primers for each gene including Bcl-2, BAD, Fas-R, caspase-3, caspase-8, caspase-9, and GAPDH as housekeeping gene were designed [10]. All of the amplification reactions were carried out in triplicate for each sample, and every experiment mixture (20 µL), containing 10 µL SYBR Green PCR master mix (Takara Bio Inc, Tokyo, Japan), 1 µL cDNA (1 µg/µL), 1 µL primer (forward and reverse), and 0.8 µL 6-carboxy-X-rhodamine (ROX as reference dye), was subjected to ABI-step I plus (Applied Biosystems, Foster City, CA, USA) instrument. One cycle at 95 °C for 5 min followed by 40 cycles at 95 °C for 20 s, 60 °C for 35 s, and 72 °C for 10 s were selected as thermal cycling condition. Pfaffle method was used for interpretation of the results and the threshold cycle values were normalized to the expression rate of glyceraldehyde 3-phosphate dehydrogenase (GAPDH) [28].

### Statistical analysis

The statistical package for the social sciences (SPSS Inc. Chicago, IL, USA version 16.0) was used for the statistical analysis. One-way ANOVA and Tukey’s post hoc test were performed for analyzing differences between all treatments and multiple mean comparisons, respectively. Statistical significance was considered to be *P* ≤ 0.05.

## Results

The IC_50_s after treatment by prepared cell-free supernatants of bifidobacteria were determined a range between 65 µg/mL to 80 µg/mL for HT-29 and Caco-2 cells at 48 h and the control group (KDR/293 cells) were treated with the highest determined concentration (80 µg/mL).

**Fig. 1:**
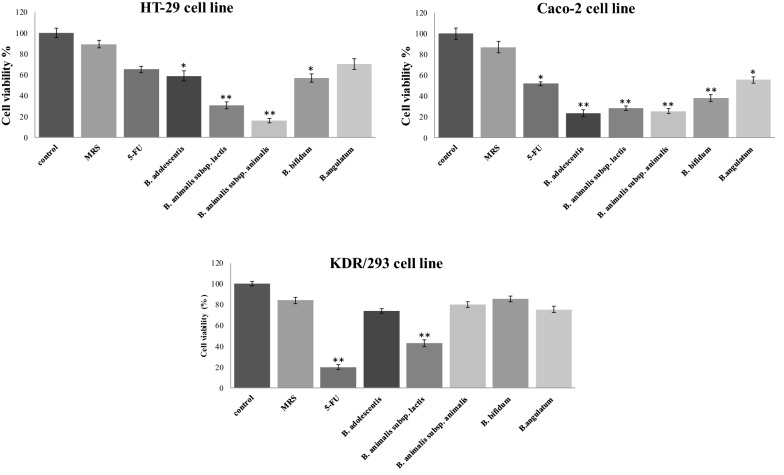
Effects of secretion metabolites of different bifidobacteria species containing indicated concentrations of dried materials from the methanolic extract on the viability of HT-29, Caco-2 cancer cell lines, and KDR/293 normal cells for 48 h incubation by MTT assay test. Untreated cells and methanolic extract of MRS media were used as negative control, and 5-FU (7 µL/well of 96-well plate) was used as positive control. Data are expressed as mean viability ± S.D. Asterisks signify statistically significant differences (^*^P ≤ 0.05, ^**^P ≤ 0.01)

After treatment by cell-free supernatant of all studied bifidobacteria species and 5-FU, the survival rates of colon cancer cells were significantly decreased in comparison to control groups. The survival rate of the HT-29 cell line after treatment by bifidobacteria was between 15.88 and 70.43% and in the Caco-2 cell line was 28.19 to 55.45%. Also, the positive control group (5-FU) showed 65.07 and 51.77% survival rates in HT-29 and Caco-2 cells, respectively (Fig. 1; Table 1).

**Table 1:** MTT assay data of treated cells from triplicate examinations and the statistical analysis in comparison to control group

Treatments	Cell lines					
	HT-29	P-value	Caco-2	P-value	KDR-293	P-value
Control	100	–	100	–	100	-
MRS	90.13	0.98	89.09	0.83	85.15	0.77
	88.19		85.67		86.51	
	89.43		85.89		84.12	
5-FU	65.21	0.77	52.12	0.03^*^	21.44	0.002^**^
	66.89		53.55		19.87	
	63.11		49.64		20.91	
*B. adolescentis*	60.47	0.04^*^	23.55	0.006^**^	75.21	0.65
	57.54		22.41		73.12	
	58.56		24.43		74.03	
*B. animalis subsp. lactis*	32.25	0.008^**^	29.45	0.008^**^	45.08	0.007^**^
	29.08		28.11		41.54	
	30.23		27.03		42.89	
*B. animalis subsp. animalis*	18.01	0.003^**^	23.05	0.007^**^	78.09	0.81
	16.45		27.08		83.14	
	13.18		26.15		80.32	
*B. bifidum*	56.38	0.03^*^	40.23	0.009^**^	87.02	0.91
	57.95		38.31		84.11	
	55.91		36.04		86.21	
*B.‌angulatum*	72.44	0.82	55.01	0.04^*^	73.08	0.79
	70.36		53.11		77.12	
	68.51		58.23		75.33	

^*^P ≤ 0.05 and ^**^P ≤ 0.01 indicate significant and highly significant increase in cell death vs. the control group

**Fig. 2:**
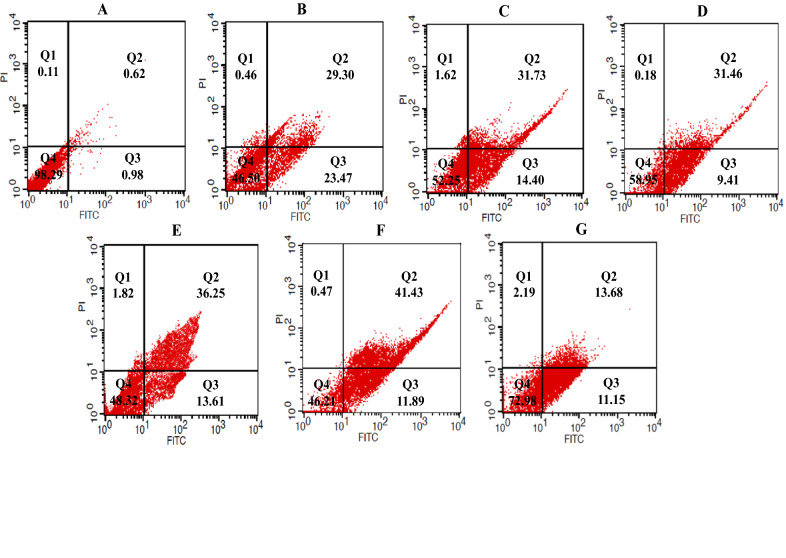
Flow cytometric analysis of treated/untreated HT-29 cancerous cells. Cells were treated with FITC-Annexin V in combination with PI to detect apoptosis and necrosis before being subjected for analysis by flow cytometry. Dots with Annexin V−/PI+ (Q1), Annexin V+/PI+ (Q2), Annexin V+/PI− (Q3), and Annexin V−/PI− (Q4) and feature represent necrotic, late apoptotic, early apoptotic, and viable intact cells, respectively. **a** Control; **b** 5-FU; **c** *B. adolescentis*; **d** *B. animalis subsp. lactis*; **e** *B. animalis subsp. animalis*; **f** *B. bifidum*; **g** *B.‌ angulatum*

**Fig. 3:**
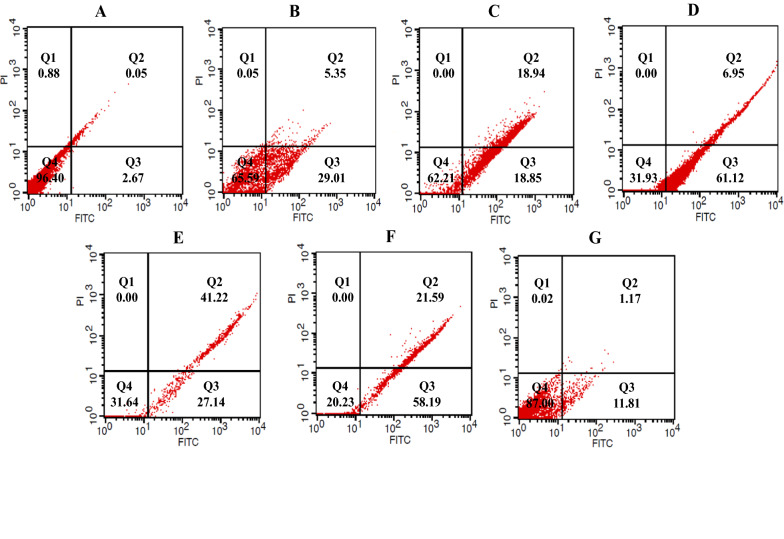
Flow cytometric analysis of treated/untreated Caco-2 cancerous cells. Cells were treated with FITC-Annexin V in combination with PI to detect apoptosis and necrosis before being subjected for analysis by flow cytometry. Dots with Annexin V−/PI+ (Q1), Annexin V+/PI+ (Q2), Annexin V+/PI− (Q3), and Annexin V-/PI- (Q4) and feature represent necrotic, late apoptotic, early apoptotic, and viable intact cells, respectively. **a** Control; **b** 5-FU; **c ***B. adolescentis*; **d** *B. animalis subsp. lactis*; **e** *B. animalis subsp. animalis*; **f** *B. bifidum*; **g** *B.‌ angulatum*

**Fig. 4:**
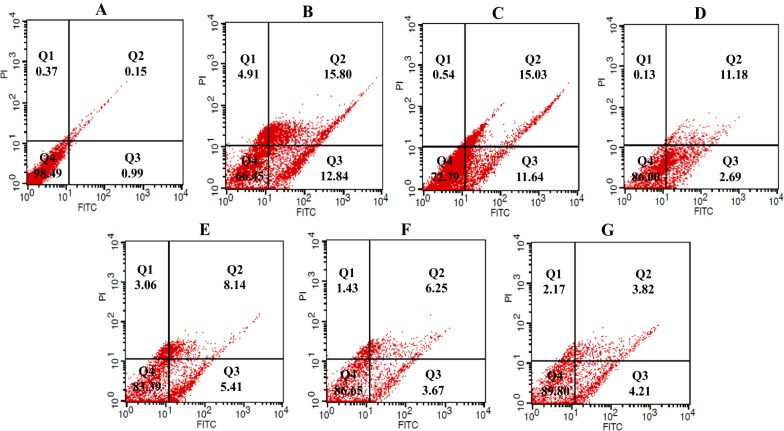
Flow cytometric analysis of treated/untreated KDR/293 normal cells. Cells were treated with FITC-Annexin V in combination with PI to detect apoptosis and necrosis before being subjected for analysis by flow cytometry. Dots with Annexin V-/PI+ (Q1), Annexin V+/PI+ (Q2), Annexin V+/PI- (Q3), and Annexin V-/PI- (Q4) and feature represent necrotic, late apoptotic, early apoptotic, and viable intact cells, respectively. **a** Control; **b** 5-FU; **c ***B. adolescentis*; **d** *B. animalis subsp. lactis*; **e** *B. animalis subsp. animalis*; **f** *B. bifidum*; **g** *B.‌ angulatum*

**Fig. 5:**
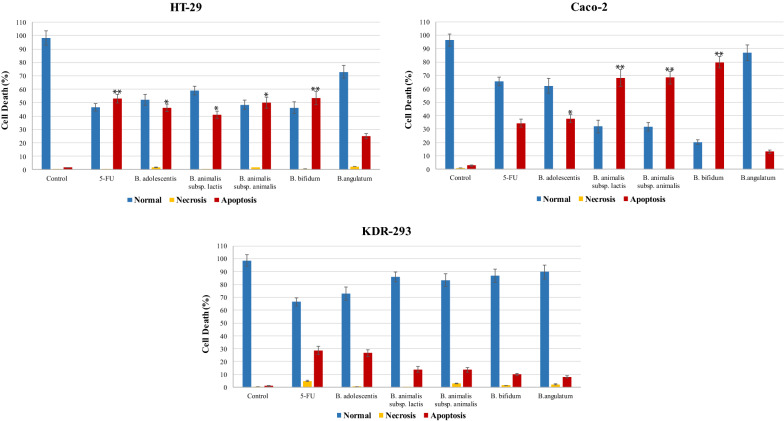
Bar plots demonstrate quantitative alterations (necrosis and early/late apoptosis) of treated/untreated cancerous and normal cell lines from flow cytometry analysis. Asterisks signify statistically significant differences in comparision to normal cells (^*^P ≤ 0.05, ^**^P ≤ 0.01)

Flow cytometry was used for the detection of apoptotic rates quantitatively with Annexin V-FITC/PI staining. According to the results of the present study, treatment of cancer cell lines with determined IC_50_ of methanolic extracts of bifidobacteria secretion metabolites increased the percentage of cells in early and late apoptosis phases compared with the control and normal KDR/293 cells. The highest percentage of induced apoptosis (early and late apoptosis) in the HT-29 cancer cell lines belonged to *B. bifidum* (53.32%) and the lowest was in *B.‌ angulatum* (24.83%) (Fig. 2). Moreover, in Caco-2 cells, the percent of apoptosis was 79.78, 68.07, 68.36, 37.79, and 12.98 in *B. bifidum, B. animalis subsp. lactis, B. animalis subsp. animalis, B. adolescentis*, and *B .‌angulatum*, respectively (Fig. 3). Also, in normal cells, the highest and lowest percentages of apoptosis were in *B. adolescentis* (26.67%) and *B.‌ angulatum* (8.03%) groups, respectively. As well, 5-FU induced 52.77% (HT-29 cells), 34.36% (Caco-2 cells) and 28.64% (KDR/293 cells) apoptosis (Fig. 4). As well, Fig. 5 showed the quantitative alterations (necrosis and early/late apoptosis) of treated/untreated cancerous and normal cells and the statistical analysis between different groups after treatment with bifidobacteria secretion metabolites (Fig. 5).

**Fig. 6:**
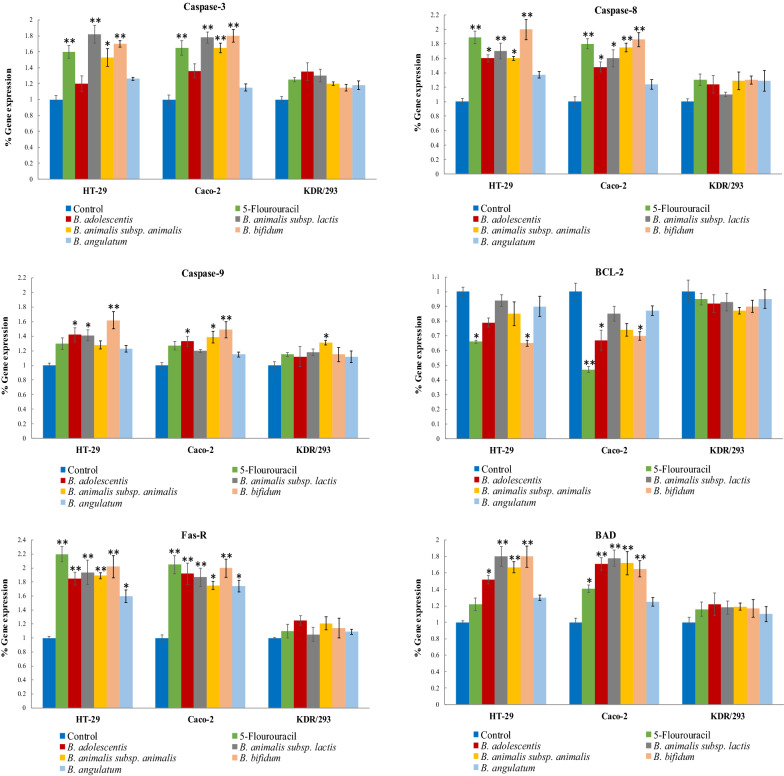
Main intrinsic and extrinsic apoptosis pathway gene expression ratio in the HT-29, Caco-2, cancer cells and KDR/293 normal cells that treated with indicated concentrations of dried materials from the methanolic extract secretion metabolites of bifidobacteria and with 5-FU as positive control group for 48 h. Target genes were normalized to GAPDH as housekeeping control gene. All experiments were performed in triplicate (n = 3) and statistic significances were accepted at *P* ≤ 0.05. ^*^P ≤ 0.05, ^**^P ≤ 0.01, as compared to control

Figure 6 indicates the expression level of pro-apoptotic (caspase-3, caspase-8, caspase-9, Fas-R, and BAD) and anti-apoptotic (Bcl-2) key genes in the colon and normal cell lines after treatment with methanolic extract of Bifidobacterial supernatants and 5-FU compared with untreated control cells. 5-FU significantly increased the expression level of pro-apoptotic genes except for caspase-9 and decreased Bcl-2 as an anti-apoptotic gene. The expression level of the BAD, caspase-3, caspase-8, caspase-9, and Fas-R genes was increased by bacterial supernatants, but only in some species was statistically significant. Treatment with *B. adolescentis* significantly up-regulated the expression level of caspase-8, Fas-R, and BAD genes in HT-29 and Caco-2 cancer cell lines. Also, the caspase-9 and Bcl-2 gene expression levels were significantly up-regulated and down-regulated in the HT-29 and Caco-2 cell lines, respectively. Moreover, *B. angulatum* could not up-regulate the expression level of all genes significantly, except for Fas-R.

## Discussion

Probiotics are being investigated for their different health beneficial effects. Anticancer or cancer-preventive effects of different probiotic species are the most beneficial properties of them and according to these findings, the consumption of probiotics or prebiotics is suggested as a promising approach for the prevention and treatment of CRC. In this way, different mechanisms have been proposed for anticancer properties of probiotics such as the production of antitumorigenic or anti-mutagenic compounds, antitoxin effects against different toxins, antimicrobial effects, production of the short-chain fatty acids (SCFA), trophic effects on enterocytes, and improvement of intestinal barrier function and microflora [11, 21, 29].

It is recently reported that probiotic microorganisms, especially *Lactobacillus* and *Bifidobacterium* species exert anticancer effects through the production of antioxidative enzymes, binding to reactive oxygen species, chelating heavy metals, and neutralizing different carcinogens. Furthermore, they can regulate the cell cycle in cancer cells and inhibit their proliferation and make them susceptible to apoptosis [13, 27, 30].

Apoptosis is a controlled process in which some defective cells are selectively eliminated and plays an important role in controlling cell numbers. Many cancer cell types including CRC are resistant to apoptosis and have uncontrolled proliferation. As well, different studies showed that bifidobacteria can interrupt this resistance via upregulation and downregulation of effective genes with pro-apoptotic and anti-apoptotic activities [30, 31]. Therefore, the regulation of cell proliferation and apoptosis can be one of the main cancer treatment strategies, and probiotics are reported to be involved in the regulation of cell apoptosis and proliferation [11, 31].

To the best of our knowledge, this is the first study that investigated the effects of secretion metabolites from different bifidobacteria species on colorectal cancer cell lines. In the present study, we investigated the potential anticancer activities of five species of bifidobacteria on HT-29 and Caco-2 cell lines. Our findings showed that the secretion metabolites of bifidobacteria species can induce intrinsic and extrinsic apoptosis pathways in human colorectal cancer cells. Also, in HT-29 cancer cell lines, the highest and lowest levels of apoptosis were induced by *B. bifidum* and *B.‌ angulatum*, respectively. The highest percentages of induced apoptosis in Caco-2 cells belonged to *B. bifidum*, followed by *B. animalis* subsp. *Lactis* and *B. animalis* subsp. *Animalis* (Fig. 5). Similarly, You et al. and Ku et al. suggested that *B.bifidum* strains inhibited the growth of several cancer cell lines including HT-29 [32, 33]. *B. bifidum* has been previously reported to be anti-proliferative and protective effects against preneoplastic lesions in animal models of colorectal carcinogenesis [8]. The anti-proliferative properties of *B. bifidum* on cancer cell lines are considered as an example of the interaction between *Bifidobacterium* spp. and host cells [34]. The interactions between *B. bifidum* and colon cancer cells lead to the suppression of cancer cell growth that indicates anticancer or antitumor effects. It is reported that whole peptidoglycan, a metabolite produced by *B.bifidum*, was capable to activate macrophages to produce large amounts of cytotoxic molecules including TNF-α, IL-6, and IL-12 [35]. By considering the antitumor activity of these mediators, the cytotoxic molecules secreted by activated macrophages mediate the antitumor effects of the whole peptidoglycan. However, in this study, the main effective metabolites of *B .bifidum* were not characterized and further studies are necessary to elucidate the main effective compound(s) and the exact role of *B. bifidum* as a preventive or therapeutic agent in CRC.

The results of the present study indicated that *B. adolescentis* can significantly inhibit the proliferation of human colon cancer cell lines, including HT-29, and Caco- 2, which is similar to the results of kim et al. [36]. Similarly, Lee et al. demonstrated that the butanol extract of *B. adolescentis* SPM0212 can decrease the proliferation of three human colon cancer cell lines, Caco2, HT-29, and SW480 in a dose-dependent manner. In addition, their findings showed that the treatment of cancer cells with butanol extract of *B. adolescentis* SPM0212 triggers macrophage activation and significantly enhances the production of TNF-α and NO as two mediators of the immune system with cytotoxic effects on tumor cells [21]. Also, Asadollahi et al. demonstrated that the treatment of LS174T cancer cells and CRC mice model by a cocktail of 5 strains of Bifidobacteria has significant protective and anti-cancer effects via downregulation of effective genes such as EGFR, HER-2, and PTGS-2 (COX-2) and suggested as the most efficient treatment in CRC [20]. Moreover, administration of live *Lactobacillus casei* ATCC 393 on murine (CT26) and HT-29 colon cancer cell lines significantly decreased the cell viability and showed potent anti-proliferative effects. Besides, the tumor-suppressive effects of *Lactobacillus casei* were associated to 60 fold higher mRNA expression of TRAIL (Tumor necrosis factor-related apoptosis-inducing ligand), as an effective gene in the activation of extrinsic apoptosis signaling pathway, and 10 fold lower mRNA expression of cyclin D1 (a protein required for progression through the G1 phase of the cell cycle), and BIRC5 (Baculoviral IAP repeat-containing 5), that encode the anti-apoptotic protein Survivin [27]. Likewise, Bibalan et al. examined the antiproliferative and anti-pathogenic effects of the Lactic acid bacteria isolated from fecal samples of healthy humans on the HT-29 cell lines. They showed that amongst the 13 *Lactobacillus* isolates, *L. plantarum* 03 has significant and maximum antiproliferative activities. In addition, they suggested that administration of a combination of *Lactobacillus* species is more effective and required for activation of the biological defense system [37].

In the present study, the investigation of underlying mechanisms revealed that the *B. adolescentis* is able to trigger apoptosis by upregulation of caspase-8, Fas-R, and BAD gene expression in HT-29 and, Caco-2 cancer cell lines. Surface receptors for extrinsic apoptosis, such as TNF-α, are produced by immune system cells and Fas, which are able to activate the cytosolic protease and caspase-8. Then, the caspase-8 activates caspase-3, caspase-6, and caspase-7 that leading to extrinsic apoptosis induction [38]. In this way, Kim et al. revealed that *B. adolescentis* enhances the production of TNF-α, as a cytokine that induces apoptosis, and their results supported our findings about the induction of apoptosis by this bacteria species[36]. Furthermore, *B. adolescentis* has been used as a vehicle for systemic delivery of the antiangiogenic protein endostatin, and systemic administration of its spores can strongly inhibit angiogenesis and reduce tumor growth [39]. Moreover, *B. adolescentis* effectively increased the expression level of the caspase-9 gene in HT-29 cells and induced the intrinsic apoptosis pathway. Caspase-9 has an important role in the intrinsic apoptosis pathway and its activation is related to mitochondrial outer membrane permeabilization and release of cytochrome *c* [40].

Furthermore, our findings revealed that *B. adolescentis* can significantly reduce the expression level of the anti-apoptotic gene, Bcl-2, in Caco-2 cell lines. Generally, apoptosis occurs via two major pathways, the intrinsic pathway (mitochondria-dependent) and the extrinsic pathways (death receptor-dependent). BCL-2 family proteins are involved in the intrinsic apoptosis pathway and composed of two groups of proteins; the first group is proteins with pro-apoptotic properties such as BAX and BAK, and the second group is proteins with anti-apoptotic properties such as BCL-2 and BCL-XL. Thus, the lower expression of BCL-2 which has anti-apoptotic activity can trigger the apoptosis pathway [40, 41].

Moreover, other species of bifidobacteria, *B. animalis*, have demonstrated anti-mutagenic activity during growth in the MRS broth which antagonizing the action of the carcinogen 2-amino-3-methylimidazo [4, 5-f] quinolone [42]. Numerous studies have focused on the potential effects of *B. animalis* strain on cancer cell lines, but the precise mechanism whereby this strain exert their antitumorigenic effects remains undetermined yet [43, 44].

In the present study, *B. bifidum*, followed by two subspecies of *B. animalis* and *lactis* exerted the highest percentage of apoptosis in the Caco-2 cell lines. Based on different studies, NF-κB has a pivotal role in inflammation and can up-regulate several genes involved in apoptosis suppression. These important effects indicate its critical role in the inflammation-related carcinogenesis [45]. It is reported that *B. animalis* subspecies *lactis* exert preventive effects on colitis-associated colon cancer by inhibition of NF-κB activity [46]. As well, Fahmy et al. revealed that treatment of CRC mice with *B. longum*, isolated from women breast milk, decrease NF-κB and IL-6 concentration. On the other hand, administration of this bacteria increased IL-1β concentration and resulted in the decline of aberrant crypt foci number in CRC-mice and improve necrosis and fibrosis of the colon cells [47]. Since the NF-κB is involved in cell proliferation and also plays a critical role in the inflammatory process, it provides a possible mechanistic link between inflammation and cancer [48]. However, we did not evaluate the activity of NF-κB, and further studies are required to investigate the effects of *Bifidobacterium* strains on NF-κB signaling.

There are some limitations to the present study. Firstly, the anticancer effects of bifidobacteria species on other types of cancer cell lines were not investigated, and additionally, the specific compound(s) of the secretion metabolites of bifidobacteria species, which is involved in the antitumor activity, was not determined. Moreover, in the present study the flow cytometry plots spilled over each other and the compensation study to separate the cell populations was not performed. Also, due to the purpose of the study and the financial constraints, the effects of Bifidobacteria species on protein levels were not investigated.

In conclusion, the present study confirmed the anticancer and apoptosis-inducing effects of secretion metabolites of bifidobacteria species on colon cancer cell lines with less adverse effects on normal epithelial cells (KDR/293). Besides, the proposed mechanisms for the CRC preventive effects of bifidobacteria species are down-regulation and up-regulation of anti- and pro-apoptotic factors. However, performing more studies is recommended to determine the exact mechanisms of probiotics in human colon cancer.

## Data Availability

The data that support the findings of this study are available from the corresponding author upon reasonable request.

## Abbreviations

BIRC5: Baculoviral IAP repeat containing 5
cDNA: Complementary DNA
CRC: Colorectal cancer
DMEM: Dulbecco’s Modified Eagle’s Medium
ELISA: Enzyme-linked immunosorbent assay
GAPDH: Glyceraldehyde 3-phosphate dehydrogenase
IC_50_: Half-maximal inhibitory concentration
IROST: Iranian Research Organization for Science and Technology
MRS: Man-Rogosa agar
MTT: 3-(4,5-Dimethylthiazol-2-yl)-2,5-diphenyltetrazolium bromide
PBS: Phosphate-buffered saline
PI: Propidium iodide
PTCC: Persian Type Culture Collection
RPMI: Roswell Park Memorial Institute medium
RT-PCR: Real-time polymerase chain reaction.
TRAIL: Tumor necrosis factor-related apoptosis-inducing ligand
5-FU: 5-Fluorouracil
